# Effects of Wild Blueberries on Fat Oxidation Rates in Aerobically Trained Males

**DOI:** 10.3390/nu15061339

**Published:** 2023-03-09

**Authors:** Kari D. Pilolla, Jessie Armendariz, Boe M. Burrus, David S. Baston, Karli A. McCarthy, Taylor K. Bloedon

**Affiliations:** 1 Food Science and Nutrition Department, California Polytechnic State University, San Luis Obispo, CA 93407, USA; kpilolla@calpoly.edu (K.D.P.);; 2 School of Applied Health, California Polytechnic State University, Humboldt, CA 95521, USA; jessiearmendariz00@gmail.com; 3 Department of Kinesiology and Sport Management, Gonzaga University, Spokane, WA 99258, USA; burrus@gonzaga.edu; 4 Core Research Facility, California Polytechnic State University, Humboldt, CA 95521, USA

**Keywords:** anthocyanins, polyphenols, wild blueberry, fatty acid oxidation, lipolysis, exercise

## Abstract

Wild blueberries (WBs) have been documented to decrease oxidative stress in active and sedentary populations as well as influence lipolytic enzymes and increase the rate of fat oxidation (FAT-ox) during rest. To examine the effect of WBs on the rate of FAT-ox and lipid peroxidation during submaximal exercise, 11 healthy, aerobically trained males (26 ± 7.5 years, 74.9 ± 7.54 kg, 10.5 ± 3.2% BF) completed a 2-week washout avoiding foods high in anthocyanins, then completed a control exercise protocol cycling at 65% of VO_2peak_ for 40 min. Participants then consumed 375 g/d of anthocyanins for two weeks before repeating the exercise protocol. WBs increased FAT-ox when cycling at 65% of VO_2peak_ by 19.7% at 20, 43.2% at 30, and 31.1% at 40 min, and carbohydrate oxidation (CHO-ox) decreased by 10.1% at 20, 19.2% at 30, and 14.8% at 40 min of cycling at 65% of VO_2peak_. Lactate was lower with WBs at 20 (WB: 2.6 ± 1.0, C: 3.0 ± 1.1), 30 (WB: 2.2 ± 0.9, C: 2.9 ± 1.0), and 40 min (WB: 1.9 ± 0.8, C: 2.5 ± 0.9). Results indicate that WBs may increase the rate of FAT-ox during moderate-intensity activity in healthy, active males.

## 1. Introduction

Attaining a higher rate of fat oxidation (FAT-ox) has various cardiometabolic benefits and when attained during exercise, endurance-based exercise performance may be enhanced. Adipose tissue, plasma triglycerides (TG), and intramuscular TGs supply fatty acids (FA) that are predominantly used for energy production during lower-intensity exercise [1,2]. As exercise intensity increases, the rate of FAT-ox decreases and cannot meet fuel demands, leading to a greater reliance on glycogen sources [1,3,4,5,6,7]. Unfortunately, sustained reliance on glycogen can result in glycogen depletion and lead to fatigue and decreased exercise capacity. Maximizing fat mobilization and oxidation and slowing glycogen depletion may improve performance at various exercise intensities [8,9].

In addition to various physical training programs, athletes are turning to dietary strategies to enhance performance, such as incorporating anthocyanins in their diet. Consumption of anthocyanins [10] has been favorable due to their anti-inflammatory and antioxidant functions [11,12]. Higher amounts of anthocyanins can be found in fruits with red, blue, and purple hues, such as wild blueberries, tart cherries, black currants, red and black grapes, and pomegranates [13]. Recent evidence suggests that the consumption of anthocyanin-rich fruits may also improve lipid mobilization and oxidation [13,14,15,16,17]. Although the specific mechanism of action is not yet determined, possible explanations include an increase in enzymatic action reducing fat absorption [18], increases in AMPK and CPT-1 activity in whole-body FAT-ox [19,20], increased transportation of FA into mitochondria [20], and increased peripheral blood flow to working muscles [21], thus increasing lipolysis and FAT-ox [2,14,17,22].

Dietary interventions aiming to maximize FAT-ox during exercise through anthocyanin-rich fruit are limited to three studies that supplemented with New Zealand blackcurrants (NZBC) [23,24,25]. Cook et al. (2015) demonstrated that 105 mg of anthocyanin from 300 mg of NZBC extract for seven days resulted in a 27% increased rate of FAT-ox via expired air sampling calculations in trained male cyclists at 65% of VO_2peak_. In their 2017 study, Cook et al. documented a significant dose effect for average FAT-ox increase of 21.5% and 24.1% from 0 mg/day to 600 mg (210 mg of anthocyanin) and 900 mg/day (315 mg of anthocyanin) of NZBC extract, respectively, in trained male cyclists at 65% of VO_2peak_. Further research with trained female cyclists consuming 210 mg of anthocyanins from 600 mg of NZBC extract for seven days also resulted in a 27% increase in FAT-ox via expired air calculations and a significant increase in non-esterified fatty acids by blood collection [25]. Wild blueberries (WB) and NZBC contain similar amounts of anthocyanins [26], although WBs are more widely available in the United States. Research on the effects of WB on FA mobilization and oxidation during exercise has yet to be conducted, as the majority of studies have focused on the health benefits of WB [27,28].

The purpose of this study was to determine the effects of a 14-day intake of whole freeze-dried WB powder on the FAT-ox rate in healthy males during a 40 min submaximal cycling bout. The objectives were to determine (1) the rate of FAT-ox during submaximal exercise using respiratory exchange ratio (RER) and substrate oxidation (FAT-ox and CHO-ox) rates and (2) the response of plasma lactate and urinary F2-isoprostanes, creatinine, and free/total carnitine during submaximal exercise. It was hypothesized that consuming 375 g/d of anthocyanins from 25 g/day of freeze-dried wild blueberry powder would result in a greater rate of FAT-ox during cycling at 65% of VO_2peak_ for 40 min.

## 2. Materials and Methods

Eleven healthy aerobically trained males (aged 26.55 ± 7.95) participated in the study (see Table 1 for descriptive statistics). Previous work examining FAT-ox rates following anthocyanin-rich fruit consumption was the basis for calculating a G *Power analysis set at a power of 80% and an α of.05 indicated a sample of 12 participants to detect a difference of 2–3% (G*Power [25]). Participants were included if they were: ≥18 years old; non-tobacco users; waist circumference < 102 cm [29]; free of any cardiovascular, metabolic, respiratory, and orthopedic conditions; not taking blood pressure and/or cholesterol-lowering medications; and not following a high fat/low carbohydrate diet. All participants engaged in moderate-intensity cardiorespiratory exercise at least three days a week for ≥ 30 min for the past two years and had a VO_2peak_ ≥ 45 mL/kg/min. All methods were approved by the Institutional Review Board at California Polytechnic University, Humboldt, in accordance with the Declaration of Helsinki.

**Figure 1:**
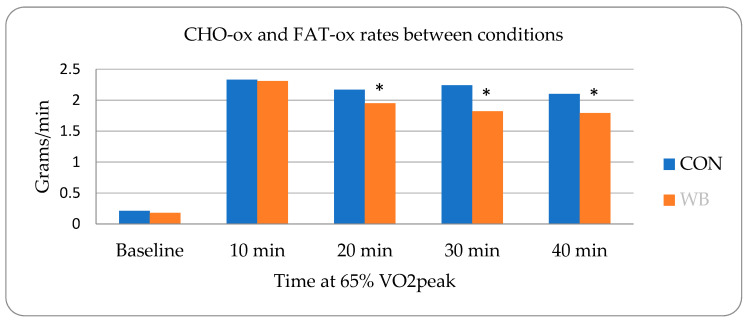
CHO-ox and FAT-ox rates between conditions. Note: * depicts significant difference (*p* ≤ 0.05).

This was a non-randomized, quasi-experimental, free-living trial. Participants were required to visit the Human Performance Laboratory at California Polytechnic University, Humboldt, at the same time (7:00 a.m.) on three separate visits (see Figure 1) over the span of four weeks and to complete two diet conditions: diet washout (C) and WB intervention. During visit 1, participants completed the consent process, health history, and diet and exercise questionnaires; had anthropometric measures taken; and then completed a VO_2peak_ protocol to determine eligibility followed by a familiarization trial. Participants were then instructed to follow the C diet for two weeks before returning for visit 2.

Two weeks after visit 1, participants returned for visit 2 at 7 a.m. after a 12 h fast following a mixed meal the night before containing carbohydrates, proteins, and fats consumed before 7 p.m. Upon consuming the meal, participants could continue to consume water in the evening and morning prior to the session. Participants were instructed to avoid strenuous exercise 48 h prior to visit 2; avoid alcohol within 24 h; avoid caffeine, aspirin, and NSAIDs the morning of; and collect and return their first-morning urine. During visit 2, once participants were seated and ready to begin, they were given a finger stick puncture to collect 300 microliters of capillary blood for later analysis. Once blood was collected, participants began warming up on the bike for 15 min, wherein the first 5 min were spent working up to 45% of VO_2peak_, and then, for the next 10 min, they slowly increased intensity until reaching 65% of VO_2peak_, which they maintained for 40 min. Immediately before, after, and every 10 min of the 40 min exercise protocol, 5–10 microliters of blood via finger stick puncture were collected to measure lactate. Immediately following the exercise protocol, participants were given another finger stick puncture to collect an additional 300 microliters of capillary blood for later analysis. Participants were allowed to cool down on the bike at their comfort level until they were ready to get off. As soon as they were able, following the exercise protocol, participants collected a second urine sample. All participants were able to collect their second sample within 15 min of completing the protocol. Following visit 2, participants were instructed to consume the WB intervention twice a day for the next two consecutive weeks while maintaining the diet washout. The third and final visit occurred two weeks after the second session when participants repeated the protocol and preparatory instructions from visit 2. Participants were instructed by trained staff to record dietary intake and physical activity during both the diet washout and WB consumption periods. Fluid intake was individualized on the basis of body weight for each subject during the two days prior to visits 2 and 3.

Diet washout: During the washout period, participants were given a list of foods to avoid and/or limit for two weeks before their second visit. This included commonly consumed foods that contain large amounts of anthocyanins such as fruits and vegetables with blue, purple, and red colors, along with red wine. Participants were also asked to avoid green tea for the remainder of the study as green tea has been shown to increase [30]. The purpose of the washout period was to ensure that any differences seen in FAT-ox the measured variables between the C (pre-supplementation) and WB conditions could be attributed to the WBs. The participants were instructed to follow a similar diet of no additional anthocyanin intake for the remainder of the study.

WB diet: Freeze-dried whole WB powder was donated by The Wild Blueberry Association in Maine containing berries from the 2017 crop. The process of freeze-drying the berry results in the least reduction in polyphenol content [31]. Participants were provided with a jar and food-grade blender ball and instructed to mix 12.5 g of the powder in 125 mL of water twice a day for two weeks. The 25 g of total daily intervention intake is equivalent to one cup of raw fruit providing 375 mg of anthocyanins and has been documented as an effective dose to decrease levels of oxidized DNA bases and increase the resistance to DNA damage [27] and is higher than the effective doses of NZBC found to increase FAT-ox in exercise [25,26,27]. The WB intervention provides 101.5 kcals, 92 g CHO, 2.15 g protein, and 15.88 g of fiber per day. Further instructions included avoiding food intake, especially dairy products, 30 min before and after WB consumption. Participants were also asked to consume the WB intervention in the mornings and evenings with a minimum of 8 h apart. Lastly, it was recommended that participants consume the WB intervention at the same time of day throughout the entire two weeks.

VO_2peak_ was determined using incremental stage protocol until volitional fatigue on a Velotron cycle ergometer (RacerMate, Seattle, WA, USA) [32]. Briefly, the protocol included a 3 min warm-up at zero watts followed by 25-watt increases every minute. After completion of the VO_2peak_ protocol, participants cooled down on the bike for 5 min before completing a familiarization trial consisting of 15 min of cycling at 65% of VO_2peak_. Given that fat is the primary fuel source during low-to-moderate intensity exercise, peaking at about 60–65% of VO_2max_, after about 15–20 min, with glucose becoming the primary fuel source as intensity increases (≥85% VO_2max_) [33], the exercise testing protocol consisted of a 15 min warm-up to reach 65% of their VO_2peak_ and sustained steady-state cycling for 40 min. Heart rate (HR), power output (measured in watts [W]), cadence, and rate of perceived exertion (RPE) using the Borg scale was measured at baseline and at 10 min intervals throughout the 40 min of cycling.

Body composition was estimated via seven site skinfold measures, namely, the tricep, subscapular, chest, midaxillary, abdomen, suprailiac, and thigh on the right side of the body using Lange skinfold calipers (Beta Technology, Santa Cruz, CA, USA). Measurements were completed in duplicate and averaged. Triplicate measures were taken when the first two skinfold measurements had a difference >2 mm [29]. Skinfold averages for each site were summed, and body density was calculated using the seven-site formula for men and was converted into body fat percentage using the Siri equation [29]. Waist circumference was measured at the upper ridge of the iliac crest using a Baseline measurement tape with Gulick attachment (Fabrication Enterprises Inc., Elmsford, NY, USA). When duplicate WC measures were > 5 mm, a triplicate measure was taken. Skinfold and WC measurements were taken at each visit to the lab to monitor changes in body composition and weight distribution.

During the C and WB intervention periods, participants were instructed to maintain and record current training regimens and daily physical activity in detail including type, duration, and intensity. Participants were also instructed to maintain current diet patterns, aside from foods designated to avoid or limit during the C and WB intervention periods, and record their diet (type, brand, preparation method, recipes, and amount of food and beverage consumed) for the three days prior to both visit 1 and visit 2. Participants were encouraged to submit pictures of their meals with their hand next to the food for serving size reference. Self-reported dietary intake and physical activity records were collected and reviewed with participants at the start of each visit. Diet records were then entered into Food Processor version 8.0 (ESHA, Salem, OR, USA) and analyzed by trained research staff; physical activity records were analyzed on the basis of total minutes/week of anaerobic and aerobic activity. Participants reported compliance with the supplementation protocol, diet, and physical activity instructions.

The primary measures included FAT-ox and CHO-ox rates using expired gases, capillary whole blood FA and glycerol, with F2-isoprostanes (≈50 mL), creatinine, and free/total carnitine (≈250 µL) via urine collection. VO_2_, VCO_2_, ventilation (V_E_), and RER were assessed with a metabolic cart (TrueOne 2400 metabolic cart, PARVO Medics, Sandy, UT, USA) [34], while rates of whole-body FAT-ox and CHO-ox were determined using the following mathematical equations with the assumption that protein contribution is minor [35]:

Fat oxidation (g/min) = 1.695 × V^ · ^O_2_ − 1.701 × V^ · ^CO_2_

Carbohydrate oxidation (g/min) = 4.210 × V^ · ^CO_2_ − 2.962 × V^ · ^O_2_

Both fingerstick whole blood and urine samples were collected pre- and post-exercise sessions and stored at −80 °C for later analysis. Glycerol and FFA were analyzed using Cayman Chemical (Ann Arbor, MI) ELISA kits at the Core Lab at Cal Poly Humboldt. F2-isoprostanes, creatinine, and free/total carnitine were analyzed using LC–MS by the Linus Pauling Institute at Oregon State University. Additional measures included lactate taken via finger puncture using the Lactate Plus Meter (Nova Biomedical, Waltham, MA, USA).

Statistical analysis was performed with JMP statistical software version 15.0 (SAS institute, Cary, NC, USA). Repeated measures analysis of variance (ANOVA) with post hoc Tukey HSD tests were used to determine differences between the dependent variables (FAT-ox, CHO-ox, lactate, urinary F2-isoprostanes, blood FA, glycerol, creatinine, free/total carnitine, RER, V_e_, heart rate, power output, cadence, and RPE) before, during, and after 40 min of cycling using condition (WB vs. CON) by time-point (pre vs. post or minutes 0, 10, 20, 30, 40). Mauchley’s test of sphericity determined the homogeneity of data, and Greenhouse–Geiser adjustments were used for violations. Differences in physical activity and food intake between the two weeks before each condition were analyzed using one-way ANOVA with pooled t-tests assuming equal variance. Mauchley’s test of sphericity determined homogeneity of data, and Greenhouse–Geiser adjustments were used for violations.

Diet records were analyzed with Food Processor version 8.0 (ESHA, Salem, OR, USA). A one-way ANOVA analysis was used to determine significant differences between total kcals, macronutrients, and antioxidant micronutrients when comparing diet between CON and WB interventions. Mixed-model analysis was used to determine if time, condition, and dietary variables influenced FAT-ox and CHO-ox responses. All data are reported in mean ± standard deviations, and significance is set at alpha level of *p* < 0.05.

## 3. Results

Data from 11 volunteers were analyzed following exclusions not meeting inclusion criteria at screening or due to injuries or non-compliance during intervention participation. Twenty-two volunteers were initially screened for inclusion in the study. Following the VO_2peak_ protocol, six volunteers did not reach the minimum 45 mL/kg/min inclusion criteria. One additional volunteer was excluded from the study due to a knee injury sustained during the WB intake period and one participant became ill before their final visit. Non-compliance to study protocols, including following a high-fat diet and not arriving for visit 3, resulted in two volunteers being dropped from the study. Lastly, one volunteer did complete all three visits; however, they became an outlier in multiple variables due to significant lifestyle changes, including substance abuse during the study, and therefore was excluded. Thus, data from eleven healthy, aerobically trained males are included in this study. No changes in body mass, waist circumference (WC), or body fat were detected in participants’ pre–post intervention (Table 1). Participants’ average total aerobic and anaerobic physical activity for the two-week period following both the control (C) and WB conditions are shown in Table 2. No significant differences between minutes of aerobic (WB: 313.3 ± 324.0, C: 325.3 ± 251.4, *p* = 0.753) or anaerobic (WB: 110.5 ± 142.5, C: 146.1 ± 160.8, *p* = 0.292) physical activity between conditions were observed.

### 3.1. Metabolic Assessments

Calculated FAT-ox rates were significantly higher following the WB condition at 20 min (WB: 0.49 ± 0.23; C: 0.38 ± 0.22, *p* = 0.049), 30 min (WB: 0.49 ± 0.26; C: 0.35 ± 0.20, *p* = 0.010), and 40 min (WB: 0.50 ± 0.25; C: 0.38 ± 0.23, *p* = 0.012) with changes of 19.7, 43.2, and 31.1%, respectively (Figure 1). These values were matched with a significant interaction between conditions over time with lower CHO-ox rates of 10.1 (WB: 1.65 ± 0.83; C: 1.84 ± 0.96, *p* = 0.0239), 19.2 (WB: 1.54 ± 0.81; C: 1.89 ± 0.96, *p* = 0.0141), and 14.8% (WB: 1.52 ± 0.79; C: 1.78 ± 0.93, *p* = 0.0451) at 20, 30, and 40 min, respectively, in the WB trials (Figure 1).

**Figure 2:**
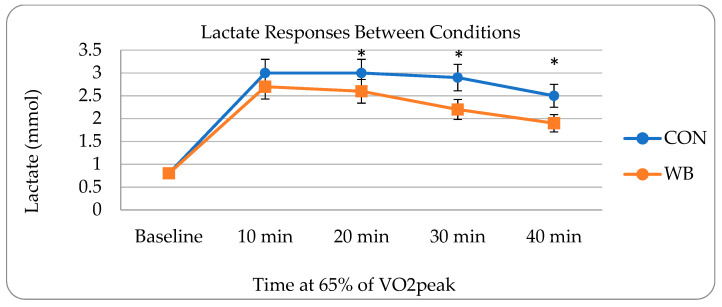
All data presented as means ± standard deviations HR (bpm), power (watts), cadence (rpm), RPE (1–10 scale), and V_e_ (L/min) lactate responses during cycling between WB and CON conditions. Note: * depicts significant difference (*p* ≤ 0.05).

There were no differences detected in V_e_, HR, power output, cadence, or RPE (Table 2) throughout the exercise sessions. Significantly lower RER values were detected over time at minutes 20 (*p =* 0.0289), 30 (*p =* 0.0097), and 40 (*p =* 0.0168) during the WB intervention. In addition, lactate values showed a significant interaction between conditions over time at minutes 20 (WB: 2.6 ± 1.0, C: 3.0 ± 1.1, *p* = 0.0053), 30 (WB: 2.2 ± 0.9, C: 2.9 ± 1.0, *p* = 0.0047), and 40 (WB: 1.9 ± 0.8, C: 2.5 ± 0.9, *p* = 0.0132) following WB consumption (Figure 2). No significant differences in urinary F2-isoprostanes, blood FFA, glycerol, creatinine, and free/total carnitine pre- and post-sessions were found during either condition.

### 3.2. Dietary Intake

Participants’ self-reported dietary intake during C and WB conditions can be seen in Table 3. Briefly, participants met ≥80% of their dietary reference intakes (DRI) for micronutrients with the exception of vitamin D, which was estimated to be 39% ± 69% and 27% ± 16% of the DRI during the C and WB conditions, respectively. There were no significant dietary differences between the C and WB conditions, with the exception of carbohydrates and vitamin B2. Carbohydrates (g/kg) and percent of carbohydrates were significantly higher in the WB intervention (*p* = 0.0043; 0.0024), with the additional 92 g of carbohydrates in the WB powder accounting for 28.35% of the total mean carbohydrate intake. Participants consumed 4.41 g/kg (56.54%) of carbohydrates in the WB intervention compared to the control condition, where participants consumed an average of 3.67 g/kg (45.63%). Vitamin B2 (mg) and percent of the DRI were significantly higher in the C condition (*p* = 0.0130). The WB powder did not add a significant amount of fat, protein, or micronutrients to the diet.

Data from the dietary analyses were further used to predict whether dietary intake would affect any physiological or anthropometric data. A mixed-model analysis, controlling for fixed effects and unobserved individual heterogeneity among participants in the form of random effects, was used to predict whether any specific dietary variable would have a significant overall effect on the output of each model. The fixed effect test of the mixed model with FAT-ox as the output response revealed that protein (g/kg) and total percent of calories from protein would significantly affect FAT-ox (*I* = 0.0074; R-squared = 0.76). Fat and carbohydrate consumption did not elicit a significant effect on FAT-ox. A secondary bivariate analysis used to predict FAT-ox revealed that vitamin A intake would significantly increase FAT-ox at the time point of 40 min during the WB condition (*p* = 0.0469).

A mixed model with the response variable as CHO-ox revealed that protein consumption (g/kg) would significantly affect CHO-ox (*p* = 0.0234; R-squared: 0.82). Two additional mixed models were used to predict body fat and WC outcomes. In both conditions, a fixed effect test revealed that vitamin A intake would significantly affect percent body fat (*p* = 0.0046; R-squared: 0.98). Additionally, on the basis of our analysis, it was found that carbohydrate intake in g/kg would significantly affect WC measurements (*p* = 0.0122; R-squared = 0.98). The output of an additional mixed model looking at RPE at 40 min suggests that vitamin A intake would significantly affect the RPE response in both conditions (*p* = 0.0031; R-squared = 0.91).

Diet analysis indicated that participants ate a minimally processed, high-quality diet throughout the study, consisting of a variety of fruits and vegetables, whole grains, a combination of animal- and plant-based proteins, and healthy unsaturated fat sources with minimal added sugars included in their diets. There were no significant differences in fruit and vegetable intake between conditions (Table 3), indicating consistent diet patterns throughout the study.

## 4. Discussion

This study was the first to investigate whether WB consumption would elicit greater FA-ox rates during an exercise protocol aimed to maximize fat oxidation using moderate-intensity exercise in healthy, trained males. Previous research examining the health impacts of fruits high in anthocyanins has found promising implications of increased FA-ox rates in sedentary individuals [16] and animals [17]. These impacts have yet to be examined with consumption of WB and the addition of exercise. Athletes, active individuals, and the general population have a growing interest in finding methods aimed at increasing FA-ox for performance, health, and body composition goals. This novel study documented that consuming WBs for 14 days increased FA-ox, decreased CHO-ox, and decreased plasma lactate values during 40 min of moderate-intensity cycling.

WB consumption resulted in significant increases in FA-ox of 19.7, 43.2, and 31.1% at 20, 30, and 40 min, respectively, following two weeks of 25 g (375 mg of anthocyanins) of WB consumption. These results vary from previous research findings with anthocyanin supplementation. In comparison to our aerobically trained participants, 20-day supplementation with Montmorency tart cherry juice (MTCJ) in recreationally active adults showed no changes in FA-ox using the same substrate oxidation calculations as used in the current study [36]. The difference in findings by Desai and colleagues (2018) may be due to the difference in fitness level and body composition of the populations as the mean VO_2peak_ was 35.87 ± 4.78 mL/kg/min compared to 54.43 ± 7.99 mL/kg/min and body fat % at 20.41 ± 10.05 compared 10.15 ± 3.37 to in the current study. The anthocyanin content of the MTCJ was not reported, and therefore comparison of dosage cannot be addressed. 

When compared to studies supplementing with NZBC on aerobically trained participants, our study demonstrated significant and greater increases in FAT-ox at all time points and overall [23,24,25]. These differences in outcomes may outline the impact of the anthocyanin concentration and variations within the fruit itself or the exercise protocol used. Cook et al. (2015) demonstrated a significant FAT-ox increase of 27% (*p* = 0.044) from minute 20–30 at 65% of VO_2max_ in a study of recreationally trained male cyclists (VO_2max_ = 53 ± 6 mL/kg/min) consuming 300 mg of NZBC (105 mg anthocyanins) for seven days. However, the differences seen in results from Cook et al. (2015) could be explained by the difference in protocol from the current study; participants in the Cook et al. (2015) study, cycled for 30 min with three 10 min increments with progressively increasing intensities at 45, 55, and 65% VO_2max_ with no differences in FAT-ox seen at 45 or 55% VO_2max_ [23]. In 2017, Cook et al. used a set cycling protocols with trained endurance cyclists (VO_2max_ = 56 ± 8 mL/kg/min) for 120 min at 65% VO_2max_ using three different doses of NZBC extract (300mg/105 mg anthocyanins, 600 mg/210 mg anthocyanins, 900 mg/315 mg anthocyanins). Results demonstrated a dose effect on increases in FAT-ox of 21.5% and 24.1% (*p* < 0.05) from 0 mg/day to 600 and 900 mg/day, respectively, with a 17.5% increase from 0 mg/day to 300 mg/day, although these changes were not significant. At all doses, there was a significant increase in FAT-ox over time (*p* < 0.001) [24]. Using the same cycling protocol as Cook et al. (2017) of 120 min of cycling at 65% of VO_2max_, Strauss et al. (2018) found a mean increase of 27% (*p* = 0.042) in FAT-ox rates in recreational trained female cyclists (VO_2max_ = 43.7 ± 1.1 mL/kg/min); using 600 mg of NZBC (210 mg anthocyanins) for 7 days. Results of this study also showed a significant increase in FAT-ox over time (*p* < 0.001) with NZBCs [25]. 

Participants in all three NZBC studies had corresponding trends in lower carbohydrate contributions during the exercise bout [23,24,25]. Although no significant decreases were found in the Cook et al. (2015) study, Cook et al. (2017) demonstrated a corresponding significant decrease in absolute CHO-ox over time (*p* < 0.001) and dose (*p* = 0.046) with no significant difference between doses, and Strause and colleagues (2018) similarly reported a significant decrease over time (*p* < 0.001) for the NZBC group, with no significant differences in the mean rate CHO-ox reduction of 12%. These results differ from the current study as CHO-ox rates demonstrated significant mean reductions at all time points, which aligns with our documented increases in FAT-ox. 

Changes in RER across all studies [23,24,25] corresponded with a decreasing trend. Cook et al. (2015) found significant decreases only at 65% VO_2max_, and in 2017, Cook and colleagues found a significant decrease in RER in both time (*p* < 0.001) and dose (*p* = 0.014) with significant mean decreases with both 600 mg (*p* = 0.014) and 900 mg (*p* < 0.05) doses. Strause et al. (2018) documented significant decreases over time (*p* < 0.001), although there were no significant differences between groups. This is similar to the current findings of significantly lower CHO-ox following WB intake indicating that participants had a greater reliance on FAT-ox. 

While each of the previous studies discussed found similar results with increases in FAT-ox and decreases in CHO-ox and RER, discrepancies between the results of the current study and of other similar studies may be explained by differences in methodology, exercise protocols, and supplementation dose. In each NZBC study [23,24,25], participants exercised two hours postprandial following a standardized breakfast consisting of porridge with semi-skimmed milk, orange juice, and a cereal bar [25], or one slice of buttered toast or bread [23,24], while the current study required participants to exercise following an overnight 12 h fast. Research has shown that exercising in a fasted state at a low to moderate intensity (<65% of VO_2max_) results in a significant increase (3.09 g) of FAT-ox without supplementation [37]. Furthermore, the intensity and total time spent exercising at a specific intensity between the current study and previous studies varied. The current study had participants cycle at 65% of VO_2max_ for 40 min, while Cook et al. (2015) had participants cycle for 10 min at three different intensities (45, 55, and 65% of VO_2maxk_) compared to the 120 min of steady cycling at 65% of VO_2max_ for both Cook et al. (2017) and Strauss et al. (2018). Despite the differences in exercise protocol, it appears that exercising for at least 40 min at 65% of VO_2max_ elicits increases in FAT-ox rates in both men and women following supplementation of an anthocyanin-rich fruit.

Another discrepancy between these studies was the dose of fruit and the corresponding dose of anthocyanins. Cook et al. (2015) used a NZBC extract containing 300 mg of fruit that contained 105 mg of anthocyanins and found no significant increases in fat oxidation at any level of intensity. It has been shown that doses of less than 100 mg of anthocyanins have little to no impact on changes in exercise-induced oxidative stress and inflammation, which may remain true for changes in substrate oxidation as well [38]. Both Cook et al. (2017) and Strauss et al. (2018) supplemented with a daily dose of anthocyanins, amounting to 210 mg of anthocyanins (300 mg NZBC extract) for 7 days with significant increases in FAT-ox. Cook et al. (2017) also supplemented with a dose of 315 mg anthocyanins (900 mg NZBC extract), although the effect size was not as strong for the 315 mg (900 mg NZBC extract) dose as the 210 mg (600 mg NZBC extract) dose (effect size: 900 mg = 0.75 and 600 mg = 1.03). The current study supplemented with 25 mg of freeze-dried fruit containing 375 mg of anthocyanins for 14 days. The higher dosage and longer anthocyanin supplementation period may be most accountable for the differences in FA-ox and subsequent CHO-ox and RER in the current study compared to past findings. Additionally, the entire matrix of the WB compared to the NZBC could be accountable for the differences observed. Discrepancies between the current study and findings in the NZBC studies [23,24,25] in FA-ox rates following anthocyanin intake may be explained by differences in methodology, exercise protocols, and supplementation dose. 

To more directly measure fat oxidation, the current study measured plasma non-esterified FAs and glycerol, with no significant differences found at rest or during exercise. Strauss et al. (2018) reported significant increases in non-esterified FAs (*p* = 0.034) and glycerol (*p* = 0.051) concentrations at rest in the NZBC condition compared to placebo; no differences were found during exercise, however. The changes in concentration of glycerol and FA in the blood documented by Strauss et al. (2018) were not replicated in the current study, which can possibly be explained by collection and analysis differences among the studies. Strauss et al. (2018) centrifuged blood samples after collection before storing the plasma only. The current study did not centrifuge the blood samples before freezing, leading to whole blood being used in the analysis. This change in the analysis was due to the availability of the initially planned analysis kit. This required a greater amount of whole blood to be used for analysis than plasma that would normally be used for the ELISA kits. This may have led to excess whole blood in the analysis kit wells and improper reading of the absorbance in the plate reader. In addition to increased reliance on FA stores during moderate-intensity exercise, there may be other benefits from WB supplementation including increasing exercise performance. Significantly lower plasma lactate values at 20, 30, and 40 min in the WB condition were found (*p* < 0.01, *p* < 0.01, and *p* < 0.05, respectively), which are similar to the results of NZBC powder intake causing a downward and rightward lactate shift at submaximal and maximal power outputs in trained triathletes [39]. Lactate removal from skeletal muscle may be impacted by increases in peripheral blood flow [21] and nitric oxide-mediated vasodilation following anthocyanin supplementation [40,41]. Increased blood flow during exercise may result in decreased mechanisms causing fatigue, such as preventing decreases in pH in the muscle and decreased force production, thus increasing exercise performance [39]. An increase in FA-ox coupled with a lower response in lactate with WBs may be beneficial to endurance exercise performance. Contrary to these findings with lactate changes, Cook et al. (2015) documented significant increases in post 16.1 km time trial plasma lactate values following seven-day NZBC supplementation in trained male cyclists. Despite these findings, Cook et al. (2015) found a 2.4% reduction in time to complete a 16.1 km cycling time trial while Murphy, Cook, and Willems (2017) found a 0.82% decrease in 4 km repeated time trials, both following seven-day intake NZBC extract, although they did not measure FAT-ox. It has been proposed that a 0.6% increase in road time-trial cycling is the smallest worthwhile change in performance [42]. Therefore, both decreases of 0.82% and 2.4% in time trial cycling from NZBC extract supplementation are beneficial to endurance performance without requiring athletes to alter their training or diet (beyond NZBC extract supplementation). An increase in FAT-ox coupled with a lower response in lactate with WB drink may be beneficial to endurance exercise performance. The current study did not include direct performance measures; however, these performance findings and differences in lactate values between conditions may have insight into WB effects on exercise performance that are currently under investigation. 

To the best of our knowledge, this is the first study to investigate the effects of WB consumption on the rate of FAT-ox during moderate-intensity exercise in humans. However, this study has several limitations. The majority of these limitations are due to the study being a free-living trial; these methods are more translational in nature, however. First, participants were entrusted to track and interpret their own physical activity and food intake during their participation. This could potentially lead to inaccurate tracking of serving sizes, exercise intensity, and duration. Second, participants were given WB powder to consume on their own time with the instructions described in the methods section, leaving room for potential error. Third, not all participants used cycling as a primary method of exercise. The study design required cycling during each session and led to some participants having difficulty determining a preferred cadence during the 40 min of cycling. Last, the exercise intensity may have been too low to see significant changes in urinary F2-isoprostanes.

## 5. Conclusions

The current study demonstrated that a 14-day intake of 25 g of freeze-dried WBs, supplying 375 mg of anthocyanins and approximately 1 cup of fresh fruit equivalent, significantly increased FAT-ox rates, decreased CHO-ox rates, and led to lower blood lactate values without changes in glycerol and FA concentrations during 40 min of cycling at 65% of vo_2peak_ in endurance-trained males. These findings may have potential effects on endurance exercise performance.

The use of anthocyanin-containing foods to increase FAT-ox during exercise has, to date, focused on the effects of NZBCs, making this the first study to investigate the effects of WBs on submaximal exercise parameters. The effects of WBs are important due to the greater accessibility in the USA compared to NZBC.

## Figures and Tables

**Table 1:** Descriptive statistics of participants (*n* = 11).

Variable	First Session	Third Session	*p*-Value
Age	26.55 ± 7.95		
Height (cm)	180.16 ± 3.42		
Weight (kg)	74.74 ± 8.22	74.91 ± 8.53	0.563
Body fat (%)	10.15 ± 3.37	10.15 ± 3.27	0.983
Waist circumference (cm)	79.41 ± 6.36	78.68 ± 5.81	0.195
VO_2peak_ (mL/kg/min)	54.43 ± 7.99		

All data are presented as means ± standard deviations *p* < 0.05.

**Table 2:** Dependent variable average comparisons between conditions at 65% of VO_2peak_.

Variable	Condition	Baseline	10 min	20 min	30 min	40 min	*p*-Value
HR	C	69 ± 10	155 ± 17	144 ± 44	162 ± 17	162 ± 17	
	WB	67 ± 7	155 ± 13	157 ± 15	160 ± 14	160 ± 14	0.434
Power	C		191 ± 24	185 ± 25	179 ± 25	177 ± 24	
	WB		190 ± 26	183 ± 27	181 ± 26	177 ± 26	0.263
Cadence	C		82 ± 6	83 ± 6	83 ± 7	85 ± 9	
	WB		77 ± 25	83 ± 10	83 ± 10	83 ± 9	0.489
RPE	C		5 ± 1	6 ± 2	7 ± 2	7 ± 3	
	WB		5 ± 1	6 ± 2	7 ± 2	7 ± 2	0.230
RER	C	0.86 ± 0.06	0.92 ± 0.03	0.90 ± 0.03	0.90 ± 0.03	0.90 ± 0.04	
	WB	0.83 ± 0.04	0.91 ± 0.03	0.88 ± 0.03	0.87 ± 0.03	0.86 ± 0.03	0.426
V_e_	C	10.9 ± 1.9	67.7 ± 7.5	68.5 ± 7.2	69.7 ± 9.1	69.1 ± 9.5	
	WB	10.0 ± 2.1	68.7 ± 8.9	66.9 ± 9.6	66.7 ± 9.6	66.5 ± 8.0	0.302

**Table 3:** Self-reported dietary intake.

	CON		DRI Met (%)		WB		DRI Met%		ANOVA
	Mean	SD	Mean	SD	Mean	SD	Mean	SD	*p* Value
Total Kcal	2450	952	75	30	2458	939	84	33	0.948
Kcal/kg	33.5	14.3	74	29	33.6	14.4	85	33	0.939
CHO (g)	270.3	23.4	64	23	324.4	69.5	82	23	0.138
Total Fat (g)	103.3	52.4	109	59	109.5	66.7	119	61	0.812
MUFAs (g)	29.7	23.3	106	80	30.9	23.6	99	68	0.906
PUFAs (g)	15.7	12.5	51	42	17.1	14	51	36	0.806
SFAs (g)	30.2	14.6	97	53	33.3	19.1	110	58	0.665
Protein (g)	106.1	43.4	184	68	105.9	31.9	169	68	0.991
CHO (%)	45.6	7.92	87	19	56.4	12.8	102	21	0.00024 *
Fat (%)	36.9	6.9	144	57	37.3	10.8	138	49	0.840
Protein (%)	17.2	4.3	106	34	18.2	3.5	103	22	0.561
CHO (g/kg)	3.67	1.4	160	47	4.4	1.2	83	23	0.0043 *
Total fat (g/kg)	1.5	0.8	112	60	1.5	0.9	117	60	0.605
Protein (g/kg)	1.4	0.6	177	73	1.4	0.5	183	55	0.917
Dietary fiber (g)	32.2	21.8	75	52	44.2	14.9	92	31	0.145
Added sugars (g)	6.1	8.6	8	12	6.5	9.4	6	9	0.914
Vitamin A (IU)	8837	7009	294	233	11,356	15,493	362	528	0.595
Vitamin E (mg)	12.1	13.6	80	91	13.4	13.1	72	43	0.429
Vitamin C (mg)	86.4	61	96	68	84.5	88.6	71	67	0.943
Vitamin D (IU)	113.7	43.4	39	69	153.9	104.1	27	16	0.263
Vitamin K (mcg)	185.4	235	154	196	326.8	763	118	179	0.551
Vitamin B1 (mg)	1.5	0.8	128	71	1.1	0.6	91	40	0.207
Vitamin B2 (mg)	2	0.9	170	96	1.1	0.6	155	70	0.0130 *
Vitamin B3 (mg)	25.2	16.4	135	101	24.9	17.9	150	112	0.982
Vitamin B6 (mg)	2.6	1.9	223	186	2.7	2.2	195	172	0.904
Folate (mcg)	433.9	1.9	108	85	409.9	323.2	89	51	0.867
Vitamin B12 (mcg)	4.3	2.4	181	99	5.3	5.6	229	227	0.602
Selenium (mcg)	87.4	49.6	158	90	93.6	25.7	174	48	0.715
Zinc (mg)	9.7	5.1	88	47	9.5	2.9	87	32	0.905
Iron (mg)	16.7	7.6	206	93	16.2	7.1	205	87	0.866

Note: DRIs for moderate PA level. CHO: carbohydrates; MUFAs: monounsaturated fatty acids; PUFAs: polyunsaturated fatty acids; SFAs: saturated fatty acids; CON: control; WB: wild blueberry; ANOVA: analysis of variance; PA: physical activity. * *p* ≤ 0.05.

## Data Availability

The data presented in this study are available on request from the corresponding author. The data are not publicly available due to specific language used in the consent form.
